# Patients’ thoughts about assessment of fracture risk in a dental setting using FRAX—a qualitative interview study

**DOI:** 10.1007/s11657-023-01259-1

**Published:** 2023-05-10

**Authors:** Charlotta Elleby, Pia Skott, Holger Theobald, Sven Nyrén, Helena Salminen

**Affiliations:** 1 Division of Family Medicine and Primary Care, Department of Neurobiology, Care Sciences and Society, Karolinska Institutet, Alfred Nobels Allé 23, Huddinge, SE-141 83 Sweden; 2 Academic Centre for Geriatric Dentistry, Stockholm, Sweden; 3 Public Dental Services, Folktandvården, Stockholm, Sweden; 4 Department of Dental Medicine, Karolinska Institutet, Huddinge, Sweden; 5 Academic Primary Care Health Centre, Region Stockholm, Stockholm, Sweden; 6 Department of Molecular Medicine and Surgery, Karolinska Institutet, Solna, Sweden

**Keywords:** FRAX, Fracture risk assessment, Qualitative study, Dental setting

## Abstract

***Summary*:**

FRAX is a tool based on questions that identifies persons at risk of fragility fractures. We interviewed patients about their thoughts on doing FRAX in a dental setting. They were generally positive but had some concerns that need to be considered before introducing FRAX in a dental setting.

**Purpose:**

To investigate patients’ thoughts about assessing the risk of fragility fractures using the FRAX tool in a dental setting. Sweden has a high incidence of fragility fractures, but many of these are preventable. The most common method for identifying persons with a high risk of sustaining fragility fractures is FRAX, a validated instrument for assessing the risk of suffering fragility fractures within 10 years. In the Nordic countries, most of the adult population has regular contact with their dentist, which could be useful in identifying high-risk individuals.

**Methods:**

A qualitative inductive approach to content analysis, with individual semi-structured interviews, was used. Seven women and three men, aged 65–75 years, were interviewed and assessed with FRAX.

**Results:**

An overarching theme was that patients considered a FRAX assessment in the dental setting a good service but doubted that the dentists would have the interest, time, and knowledge to do it. The patients had little knowledge and experience of osteoporosis and fragility fractures. They were positive towards assessing the fracture risk with the FRAX instrument. If they were found to have a high fracture risk, they expected the dentist to send a referral for further investigation and to collaborate in the risk assessment with their family physician. They thought risk assessment in a dental context would be a good service if the fee was the same as that in primary care.

**Conclusion:**

Most participants were positive about having FRAX and other health assessments done in the dental clinic, but this study shows that patients have concerns that need to be addressed before introducing FRAX in this context.

## Introduction

Fragility fractures, i.e. fractures of the hip, wrist, upper arm, and pelvis, are associated with frailty in older persons and can result in the need for extensive care, a place in a nursing home and even death. Sweden has among the highest incidences of fragility fractures in the world, but many of them could be prevented with medication and fall-preventive measures if the persons with high risk are identified [1]. The most common method of identifying persons with a high risk of sustaining fragility fractures is the FRAX tool [2]. It is a validated instrument based on large cohorts from different countries used to assess the risk of fragility fracture within 10 years [3]. It uses an algorithm including risk factors such as age, sex, BMI, previous fractures, heredity, and smoking. The FRAX risk score can be used to guide health care if further handling of the patient is needed. For example, the Swedish National Board of Health and Welfare recommends antiresorptive medication if the FRAX risk score of a major osteoporotic fracture exceeds 30% [4].

FRAX has been used in a dental setting in previous studies but only to find associations with dental diagnoses. The association between osteoporosis and dental health has been studied from different angles, with or without using FRAX. Several studies have focused on the association between chronic periodontitis and osteoporosis, and systematic reviews indicate that postmenopausal women with osteopenia or osteoporosis have more severe periodontitis [5, 6]. Higher FRAX scores have been associated with impaired periodontal status in postmenopausal women [7], and Penoni et al. also found in a later study that periodontal disease was not a predictor for osteoporosis, although FRAX was “a useful tool to suggest periodontal evaluation” [8]. In another study, Priebe et al. found an association between the number of missing teeth, hip fractures, and a FRAX assessment of fracture risk [9].

In the Nordic countries, most of the adult population has regular contact with their dentist, which could be used to try to prevent fragility fractures in connection with a scheduled check-up. Features on dental radiographs, such as the trabecular pattern and width of the mandibular cortex, are associated with low bone mass density (BMD), and these have been suggested as useful for finding patients at risk of fragility fractures. Intraoral radiographs have been used for both visual and semi-automated assessments of the trabecular pattern of the mandibular bone [10–12]. The visual method is operator dependent and requires careful calibration, and there is no semi- or automated method that uses intraoral radiographs with sufficient scientific evidence to justify individual risk assessment [13]. Studies using panoramic radiographs have included different aspects of the morphology of the mandible, such as the cortical width and porosity, and trabecular structure, often using different indexes, to identify patients with a higher risk of osteoporosis or fragility fracture [14–17]. However, panoramic radiographs are not available in all dental clinics; they are also more expensive and imply higher doses of radiation than intraoral radiographs. In contrast, the FRAX tool offers a validated, inexpensive, and frequently used method to find patients with a high risk of fragility fractures. Before suggesting the use of the FRAX tool in a dental setting, there are important aspects to be investigated such as what patients, and dental and medical staff, think about this. Qualitative studies of methods for medical risk assessment in a dental setting have been made, but none using FRAX. Gullberg et al. used focus groups to study the implementation of a method of risk assessment for osteoporosis by evaluating the trabecular pattern on intraoral radiographs [18]. The focus groups included both dental and medical staff as well as persons from patient support groups. The latter expressed a lack of knowledge of osteoporosis, both among the public and medical professionals. They saw screening for osteoporosis at a regular dental visit as positive for both the patient and society and expressed a need for referral to medical care if the risk was high. The willingness to pay for an osteoporosis risk assessment was also studied [19], where a majority were willing to pay, with a mean of about 45 €. Most participants found it valuable if dental clinics would offer osteoporosis risk assessment. Friman et al. studied screening for cardiovascular disease and diabetes in a dental setting, including a qualitative study with patient interviews [20, 21]. Their participants appreciated medical screening and thought it important that the dental staff possesses sufficient medical knowledge. They also emphasised the need for good cooperation between dental and medical care, with referrals if necessary. In a British study using questionnaires, most of the patients had a very positive attitude towards chair-side medical screening in the dental setting [22].

General health screening is not done on a general basis in Swedish dentistry, and there is no register for this. There are several research projects going on in Sweden using the dental appointment to identify patients with high blood pressure, high blood glucose, or risk for pre-diabetes. In Sweden, private practitioners treat about 2/3 of the adult population. Some of these offer assessments of blood pressure and blood sugar as a service to their patients. Except for the previously mentioned studies by Friman et al. [20, 21], there are so far no published studies or evaluations of these services. Some Swedish private dentists have previously offered an assessment of the risk of osteoporosis by uploading intraoral radiographs on a website together with some information about gender, age, etc. The trabecular pattern of the bone on the radiograph was assessed over several years by a private company, and this resulted in a high, medium, or low risk of osteoporosis, although the scientific evidence supporting this method of assessing fracture risk on an individual level was poor [12, 13]. Some pharmacies in Sweden offer a measurement of blood pressure or blood sugar, often in collaboration with a private primary health care provider. However, these data are not easily shared between the different medical professions, e.g. doctors, dentists, and pharmacies, unless referrals are made. Primary care physicians never have access to dental records, for instance, even though many patients think that they do. This leaves the patient responsible for any action due to the screening result, which may inhibit the good intentions associated with the screening. This qualitative study aimed to explore the thoughts of dental patients about assessing the risk of fragility fractures using the FRAX tool in a dental setting, for example, in conjunction with a regular check-up.

## Material and methods

### Design

In this study, we used the qualitative research design of content analysis, as described by Graneheim and Lundman, with face-to-face, individual, semi-structured interviews using an interview guide [23]. The inductive approach was used because we wanted to freely explore the participants’ thoughts, which have not previously been studied.

### Participants

The participants were recruited by asking fellow dentists at three separate clinics of public dentistry: one in Jakobsberg (a suburb of Stockholm) and two in the centre of Stockholm (Stockholms Sjukhem and Fridhemsplan). Those patients that fulfilled the inclusion criteria were approached and asked if they would participate in the study. The dentists then sent the names and telephone numbers to the first author (CE) who contacted the patients. Only one person did not respond or declined to participate when contacted. The inclusion criteria were a patient at Stockholm Public Dentistry aged 65–75 years. Only persons with Swedish ethnicity were included because the FRAX assessment depends on the country of origin. To avoid bias, we excluded patients who were, or had been, patients of CE or the observer co-author PS (both dentists). No medical doctors, medical nurses, or dentists were included in the study as we did not think that they would present the thoughts of an ordinary dental patient. One patient was found to be too young to fulfil the inclusion criteria and was therefore excluded. We interviewed participants until we reached saturation, and new interviews did not add new content. This resulted in a total of ten interviews.

### Data collection

Ten interviews were conducted from February 25 to May 9, 2022, of which four took place in a room adjacent to the Clinic of Public Dentistry in Jakobsberg and six in a room adjacent to the Clinic of Public Dentistry at Stockholms Sjukhem. All interviews were performed by CE, with author HS as an observer at three interviews and PS as an observer at four. Three interviews were given without any observer present. We used the same interview guide for all ten interviews, and these ranged from 18 to 45 min in duration (see Appendix). Field notes were taken by the observers and discussed after the interview.

### Data analysis

In the analysis, an inductive approach was used, identifying the manifest and latent content of the interviews described by Graneheim and Lundman [23]. The interviews were audiotaped and transcribed verbatim by CE. Only CE had access to the participants’ identities during the analysing process, but the categorisation was done without access to the participants’ identities for all researchers. The analysing group consisted of CE, HS, PS, and HT. The transcripts and notes taken during the interviews were read repeatedly to capture their essence. Relevant meaning units, i.e. phrases meaningful to the aim of the study, were first identified, then condensed, and later made into codes. This was done with half of the interviews by HS, PS, and author HT individually; the rest were coded by CE and HS. The analysing group discussed the choice of meaning units and the codes until consensus was achieved. We then compared the codes, and those with similar content were classified into separate subcategories. All authors discussed, regrouped the codes, and renamed the subcategories until consensus was once again achieved. In the next step, we looked for similarities in the subcategories and found three categories into which all subcategories would fit. Representative quotes from the interviews in all subcategories were chosen to illustrate the manifest content of the interviews in each subcategory. Finally, we discussed and together identified an overarching theme that reflects the latent content present in most categories. To ensure that saturation was obtained, we checked that the last two interviews did not contribute any new ideas or categories. This comprehensive description of the analysis process is intended to increase the study’s dependability.

### Ethics

The Swedish Ethical Review Authority approved this study on November 29, 2021 (Dnr 2021–05,900-01). All procedures involving human participants performed in the study were in accordance with the ethical standards of the institutional and/or national research committee and with the 1964 Helsinki Declaration and its later amendments or comparable ethical standards. All individual participants included in the study were informed in writing and orally before they gave their written informed consent about the study and their right to decline to participate.

## Results

We recruited seven women (65–74 years old) and three men (65–75 years old), with a mean age of 70 years (Table 1). Most participants were retired, while a few were still working, although not full-time. The participants had different social backgrounds and educational levels, although this was not an expressed goal in recruitment. (One participant was a former dental nurse and supervisor who was briefly acquainted with CE and had worked with PS earlier). The 10-year risk of a major fragility fracture, according to the FRAX assessment, ranged from 6 to 34%. One participant, who was found to have a risk > 30%, was recommended to contact primary care.

**Table 1:** Characteristics of the participants

	Women	Men	Total
Sex (n)	7	3	10
Age in years, range (mean)	65–74 (70)	65–75 (70)	65–75 (70)
FRAX, 10-year risk of major osteoporotic fracture %, range (mean)	8–34 (19)	6–25 (13)	6–34 (17)

The analysis resulted in three main categories with up to nine subcategories (Table 2).

**Table 2:** Main categories and subcategories

Experience and knowledge of osteoporosis and fragility fractures
The participants’ own experiences
Knowing others with experience of osteoporosis and fragility fractures
Knowledge about osteoporosis and fragility fractures
Thoughts about the FRAX assessment
How the FRAX assessment was experienced
Thoughts about the assessment result
Perception of risk
Actions after the result
Risk assessment in a dental context
Doing the FRAX assessment in the dental setting
FRAX assessment as a screening method
A preventive perspective on FRAX assessment
Paying for the assessment
Handling of the FRAX assessment result
Structural problems
The dental staff’s engagement and competence
Other contacts with health care
Other health assessments in a dental setting

An overarching theme was that patients think offering FRAX in the dental setting is a good service, but they doubt if the dentists have the interest, time, and knowledge to do it.

### Experiences and knowledge of osteoporosis and fragility fractures

Among the participants were those who had thought of osteoporosis before and those who had had no thoughts or experience of fragility fractures. Even though one had fallen and had a radius fracture, she did not consider it to be a fragility fracture. “I was doing my Nordic Walking, and then he came cycling right at me. He was looking at his mobile phone, and I fell forwards with my poles, and hit the ground like this, resulting in a small radius fracture. It was just an accident, it could have happened to anybody when getting hit from behind, even for someone who doesn’t have osteoporosis” (#9, female). Another participant had had a heel scan because her older female relatives had lost height late in life, while still another had had a DXA scan because of corticosteroid medication, but neither were found to have osteoporosis.

Several participants had relatives or friends who had fallen and had fractures, but few thought of this as a fragility fracture. “My mother fell when she was 80 and broke her hip, but I don’t think that was because of osteoporosis” (#4, female).

The females who had reason to suspect they could have osteoporosis, hereditarily or because of medication, had more knowledge about osteoporosis than others. The males had not thought at all about osteoporosis. As one participant said, it is not something you discuss among peers: “It’s not something you talk about in everyday life when you meet someone: -Have you got osteoporosis? No, to be honest, you mainly talk about (osteo)arthritis, which I have had knee replacements for” (#4, female).

In conclusion, the participants had little knowledge of osteoporosis and fragility fractures and had never heard of FRAX. Even if they had relatives who fell and broke a hip or became shorter at an advanced age, they did not think it was because of osteoporosis.

### Thoughts about the FRAX assessment

The FRAX assessment was found to be easy and simple, and it was appreciated that it only meant answering questions and did not involve more invasive methods, e.g. a blood sample.

“It’s like filling in (the health declaration) when you have the vaccine, it’s the same thing. (#4, female).

Before the assessment, the participants thought they would be very happy if the assessment would show low risk, even though they did not expect the risk to be high. “That’s roughly what I would expect the result to be, yes, exactly” (#8, female). Some stated that if they would be found to have a high risk of fragility fracture, they would be worried but at the same time glad they found out so they could do something about it. “I would probably find it hard to find out, but not too much. At the same time, I would be pleased I found out” (#3, female).

After an assessment with a “low risk” result, they admitted they were happy and relieved*.* “It feels very good, of course, you feel happy when you get positive results” (#7, male).

The only participant found to have a high risk of having a major osteoporotic fracture according to FRAX (> 30%) was very surprised as she, despite a few previous falls, had still not broken anything. “I was slightly surprised that I was at such high risk. I actually didn’t think so. Considering I have had a few falls and that was all there was. (#9, female).

Some of the participants were aware that the risk of fragility fractures rises with age and that exercise can be preventive. “It’s deceptive, and the older you get, the greater the risk that you stumble. You don’t even need to be that old, because when you reach my age, in your sixties, you can easily miss a step on the stairs and all that. That’s happened several times, so you can easily fall” (#3, female). If the result would show a high risk, the participants thought they would be eager to learn more about what they could do to prevent fractures and mentioned that they would exercise more and be increasingly cautious when moving around.

“I would probably do something actively myself, not just sit and watch. I would probably be more careful, and possibly exercise in a more balanced way, both my balance and how I exercise” (#9, female). They also expected to have contact with someone like the family doctor/GP to find out more.

“I could turn to my doctor (your GP?) yes that’s right. I think I would try to find out what more I could do to prevent it from getting worse” (#3, female). There was also a feeling of pragmatism and acceptance of the result if the risk was high. They would have to accept it and do whatever they were advised to avoid fragility fractures.

In conclusion, the FRAX assessment was found to be easy and simple, and most participants were interested to know the results. Most of the participants were not worried about having a fragility fracture, but some exercised and kept in shape to prevent falling and having one. If the assessment would have showed a high risk, some said they would have become more cautious and followed the advice on physical training.

### Risk assessment in a dental context

Several expressed that they would appreciate it if the FRAX assessment was offered at the dental clinic at a scheduled check-up, although one person had some reservations: “No, the dentist should probably do their thing. We do after all have the Primary Care (for this)” (#4, female). There was a feeling of surprise that the dental staff was interested in the whole body, not just the teeth. “Yes, slightly unexpected, but positive, in my opinion. At least you go to the dentist pretty regularly” (#8, female).

As osteoporosis exhibits no symptoms before a fracture, they thought an assessment would be favourable. “It’s good to do something like that and check if you are at risk of osteoporosis and to do it early somehow…so you don’t walk around not knowing you have it. It doesn’t show, so to speak, not until you break something, that’s how I’ve understood it” (#3, female). There was a suggestion that when you reached a certain age, this would automatically mean there would be an offer to do a FRAX assessment in the dental clinic. “In that case, you should get called (to the dental clinic) for a compulsory assessment when you reach a certain age” (#2, male).The participants thought that a FRAX assessment could prevent fractures because if you find that you have a high risk, you could prevent fractures with, for example, training and diet. “If you are prone to osteoporosis, you could at least prevent the risk with a little physical training, to keep in shape and not just sit on the couch, but maybe do exercises instead, or whatever, because then you know that if you do this, maybe you can postpone the onset a bit.…” (#4, female).

When it came to paying for the FRAX assessment, the participants were generally unwilling to pay more than it would cost to have it done in primary care (about 25 Euros) “I have no problem with that (paying for a health care assessment) as long as the cost is reasonable…like an appointment at the health care centre” (#8, female).

If the FRAX assessment showed a high risk, the participants would have liked advice on how to handle it, either directly from the dentist or through a referral to primary care. “I would expect some kind of collaboration between dental care and primary care, so I would know how to go further. Maybe they could send an immediate referral or something like that… Don’t leave me with the result, help me get on with it!” (#1, female).

There was a feeling that dental care is separated from the care for the rest of the body and a lack of comprehension as to why this is so. “I really don’t understand why dental care and primary care are as separated as they have been” (#1, female).

The participants would only agree to do a FRAX assessment if they trusted the dentist, but some questioned if the dental staff really has the knowledge and time and is interested in doing it. “I guess it’s a good addition to get done at the dentist. At the same time, I wonder if there really is time for that. Are dentists interested? Or, well, they have their time with… dental care takes time and all that. So, I really don’t know how that would be solved” (#3, female).

Those who already had regular contact with health care because of chronic illness were less likely to be positive about doing health assessments at the dental clinic. They also wished that the dental or health care office would take the initiative to do health assessments. “That depends on how much contact you have with health care. Personally, I have very frequent contact in various health care settings, so I’m sure I would get a referral if something was detected” (#2, male).

Some thought it is a really good idea to do other health assessments in the dental clinic, at least if they have something to do with oral health. “There is so much that can be seen in the mouth… and if you can have other types of assessments included, that would be very positive, absolutely!” (#6, female). Nevertheless, others thought health assessments should be restricted to health care staff only.

In conclusion, most participants were positive about having FRAX and other health assessments done in the dental clinic, at a cost not exceeding what could be expected if done in primary care. If they were found to have a high risk, they expected some collaboration with, for example, their family doctor/GP for further investigation and advice.

## Discussion

We found that the patients were generally positive about doing the FRAX assessment in the dental setting. They had little knowledge of osteoporosis or fragility fractures unless they had risk factors such as corticosteroid medication and had been informed by their doctors. While they thought it would be a good service to do the FRAX assessment at the dental clinic, some doubted that the dental staff had the knowledge or the interest for it. Most participants were willing to pay for the assessment, but not more than what it would cost in primary care.

Some of the participants were aware that fragility fractures could be prevented, mainly by avoiding falling. Exercising was also mentioned, both to obtain a better balance and muscular strength. However, none of the participants mentioned medication as a method to prevent fractures, although medication with both bisphosphonates and denosumab is known to have a good effect, with a decreased risk of new vertebral fractures of about 40–70% [24, 25].

Our results are in line with another qualitative study, made in preparation of implementing a method of risk assessment for osteoporosis evaluating the trabecular pattern in intraoral radiographs to predict fracture risk [18]. They interviewed focus groups including both dental and medical staff, as well as persons from patient support groups. The latter had usually joined the support group after having been diagnosed with osteoporosis, often after several fragility fractures, and they were all female. Since they suffered different levels of impairment due to these fractures, they expressed that they would have liked to have had the diagnosis before the fractures had occurred. It is possible that they were biased towards any fracture risk assessment because of their experience of osteoporosis or fragility fractures. In a quantitative study using questionnaires, Greenberg et al. found that patients had a positive attitude towards chair-side medical screening in a dental setting [26]. Their participants ranked the fact that the assessment was not done by a physician as the least important potential barrier to the implementation of medical screening. Performing the screening of the individual participants, while investigating their thoughts about it, could influence their thoughts or attitudes, but studies have been conducted both with and without screening the participants, and the results are similar. Previously mentioned studies by Creanor et al. and Greenberg et al. found the participants to be positive towards medical screening in a dental setting without having undergone the screening itself [22, 26]. In the study by Friman et al., and a Practice-Based Research Network study in the US and Sweden, the screening method was used in conjunction with the interviews/questionnaires, and they also found that patients had positive attitudes towards medical screening in a dental setting [21, 27]. The risk of causing feelings of uncertainty and worry by just informing of the risk of osteoporosis, as studied by Hvas et al., was not confirmed in this study [28]. Our participants were more pragmatic and thought they would be happy to discover whether they had an elevated risk of fragility fractures so they could prevent fractures through training.

The strengths of this study include that the participants were representatives of regular patients at the public dentistry. This increases the credibility of the study. We excluded medical doctors and dentists as patients because their role as clinicians probably overrules their role as a patient. However, we had one dental assistant/nurse and supervisor, as well as one radiology nurse, both of whom were still working part time. They had slightly more knowledge about osteoporosis and fragility fractures and had more traditional views concerning who should do what in risk identification. The researchers in this study have different professions (three medical doctors and two dentists), and all have different clinical working fields and different research experiences. This also contributes to the credibility of the study by way of researcher triangulation in the analysis process. The interviews went smoothly, perhaps because the interviewer has over 30 years of experience communicating with patients as a dentist.

A limitation of the study may be that since the FRAX instrument, which was developed about 15 years ago, uses a binary categorisation for sex as a risk factor, we also used this in our study. Another limitation with the study is that we only included participants with Swedish ethnicity. This was done because the validation of the FRAX instrument was done on a Swedish population. Moreover, the fact that the interviewer is a dentist, which was known to all participants, may have influenced their responses and therefore be a further limitation of the study. The interviewer’s background as a dentist may also have influenced her interpretation and analysis of the interviews. On the other hand, being familiar with the dental setting may facilitate the research and increase credibility. As the patients that were asked by the dentists to participate were probably partly selected for their presumed willingness to participate and their ability to communicate, this could result in a selection bias of the cohort. However, we are dependent on the ability to participate and communicate so this is most likely inevitable.

## Conclusion

Although the participants had little knowledge of osteoporosis or fragility fractures, most of them were positive about having FRAX and other health assessments done in the dental clinic, at a cost not exceeding that charged in primary care. However, some of them doubted that the dentists would have the interest, time, and knowledge to do it. If they were found to have a high risk, they expected the dentist to collaborate with their family doctor/GP for further investigation and advice.

The ageing of the population, with added years of health but also frailty, may imply more suffering for the affected patients and increased costs for society. To meet this challenge, it may be necessary to use all possible ways of medical screening to find high-risk individuals, and the dental setting may be one of these. FRAX may be a useful tool in the right hands, but this study shows that there are thoughts among patients to bear in mind before considering introducing it there.

### Supplementary information

Below is the link to the electronic supplementary material.

Supplementary file1 (DOCX 17 KB)

## Data Availability

The original data, which is in Swedish, can be accessed by contacting the corresponding author.
