# Supplementary material for: Patients’ thoughts about assessment of fracture risk in a dental setting using FRAX—a qualitative interview study
Source: Arch Osteoporos. 2023 May 10;18(1):65. doi: 10.1007/s11657-023-01259-1 (PMC10172282; doi:10.1007/s11657-023-01259-1)
Supplement: Supplementary file 1 — Supplementary file1 (DOCX 17 KB) [file 11657_2023_1259_MOESM1_ESM.docx]

Supplement, Archives of Osteoporosis

**Patients' thoughts about assessment of fracture risk in a dental setting using FRAX - a qualitative interview study,**

Charlotta Elleby, Pia Skott, Holger Theobald, Sven Nyrén, Helena Salminen

Corresponding author: Charlotta Elleby, [charlotta.elleby@ki.se](mailto:charlotta.elleby@ki.se)

Karolinska Institutet, Department of Neurobiology, Care Sciences and Society

**Interview guide study IV – translated from Swedish**

**Interview themes:**

- The interviewees’ knowledge of osteoporosis
- Using instruments to identify fragility fractures, e.g., FRAX
- The interviewees’ own risk to have a fragility fracture

**Introduction:**
This interview is going to be about fragility fractures. If you have osteoporosis there is an elevated risk to have so called “fragility fractures”, i.e., fractures of the hip, arm, wrist, or vertebrae that occur even when the trauma is small, like when you trip and fall on the floor, or lift something heavy. The risk is generally greater for women than men and rises with age. Every other woman and every fourth man in Sweden are predicted to have a fragility fracture during their lifetime.

**Interview questions:**

- If you, or someone you know, has experience of osteoporosis or broken something because of osteoporosis, can you tell me more about that?
- There is a tool called FRAX that consists of simple questions that you can fill in to calculate the risk of having a fragility fracture within the next ten years (You state sex, age, weight, height, if a parent has had a hip fracture, corticosteroid medication etc.)
  - What do you think when you hear that?
  - How do you experience the perspective of ten years?
- How would you experience an offer from your dentist to do a FRAX test to assess your risk of having a fragility fracture during the next ten years?
  - What would make you accept or decline the offer?
  - Which feelings do you think you would have if you had a high risk?
  - Which feelings do you think you would have if you had a low risk?
  - How do you expect the result to be handled?
    - What would you like the dentist to do with the result?
    - What would you do yourself when you knew the answer?

**The FRAX assessment** is done of the interviewee by the interviewer

- Now that we have done the FRAX assessment, what was your experience of it?
  - Describe how you feel and think about the result we acquired.
  - How do you view having done the assessment in a dental clinic?
  - If the dentist (?) would offer more assessments like this, let’s say measuring the blood pressure or some other test that concerns your general health and not exactly your oral health, what do you think about that?
  - What do you think now, after having done the assessment, about paying for a FRAX-assessment at the dentist?
  - What do you think about paying for another health assessment, for example measuring your blood pressure, at the dentist?
